# Crypt and Villus Transcriptomic Responses in Mouse Small Intestine Following Oral Exposure to Hexavalent Chromium

**DOI:** 10.1093/toxsci/kfab152

**Published:** 2021-12-22

**Authors:** Grace A Chappell, Jeffrey C Wolf, Chad M Thompson

**Affiliations:** 1 ToxStrategies, Inc, Asheville, North Carolina 28801, USA; 2 EPL, Sterling, Virginia 20166, USA; 3 ToxStrategies, Inc., Katy, Texas 77494, USA

**Keywords:** dose-response, gene expression, metals, molecular mechanisms, omics research, RNA-seq

## Abstract

Oral exposure to hexavalent chromium (Cr(VI)) induces tumors in the mouse duodenum. Previous microarray-based transcriptomic analyses of homogenized mouse duodenal tissue have demonstrated Cr(VI)-induced alterations in various cellular pathways and processes. However, X-ray fluorescence microscopy indicates that chromium localizes primarily to the duodenal villi following exposure to Cr(VI), suggesting that previous transcriptomic analyses of homogenized tissue provide an incomplete picture of transcriptomic responses in the duodenum. Herein, transcriptomic analyses were conducted separately on crypt and villus tissue from formalin-fixed paraffin-embedded transverse duodenal sections from the same study in which microarray-based analyses were previously conducted. A total of 28 groups (7 doses × 2 timepoints × 2 tissue compartments) were analyzed for differential gene expression, dose-response, and gene set enrichment. Tissue compartment isolation was confirmed by differences in expression of typical markers of crypt and villus compartments. Fewer than 21 genes were altered in the crypt compartment of mice exposed to 0.1-5 ppm Cr(VI) for 7 or 90 days, which increased to hundreds or thousands of genes at ≥20 ppm Cr(VI). Consistent with histological evidence for crypt proliferation, a significant, dose-dependent increase in genes that regulate mitotic cell cycle was prominent in the crypt, while subtle in the villus, when compared with samples from time-matched controls. Minimal transcriptomic evidence of DNA damage response in either the crypts or the villi is consistent with published *in vivo* genotoxicity data. These results are also discussed in the context of modes of action that have been proposed for Cr(VI)-induced small intestine tumors in mice.

The oral carcinogenicity of hexavalent chromium (Cr(VI)) was demonstrated in a 2-year cancer bioassay in rats and mice (NTP, 2008; Stout *et al.*, 2009). Epidemiological evidence for oral carcinogenicity of Cr(VI) is weak or nonexistent (Gatto *et al.*, 2010), likely due to the fact that, based on the animal data, the oral carcinogenicity of Cr(VI) requires prolonged exposure to levels that are orders of magnitude above typical environmental concentrations. Specifically, median and 95th percentile Cr(VI) levels in U.S. water sources are 0.001 and 0.003 ppm, respectively (McNeill *et al.*, 2012; Moffat *et al.*, 2018; Oze *et al.*, 2007; U.S. EPA, 2017), whereas oral carcinogenicity in the mouse small intestine occurs at ≥30 ppm and in the rat oral cavity at 180 ppm (NTP, 2008; Stout *et al.*, 2009). Multiple groups have proposed modes of action (MOAs) for Cr(VI)-induced carcinogenicity, including genotoxic (McCarroll *et al.*, 2010; Zhitkovich, 2011) and nongenotoxic MOAs (Thompson *et al.*, 2013, 2017a). More recently, an adverse outcome pathway for chemical-induced intestinal carcinogenesis via nongenotoxic key events in mice has leveraged Cr(VI) data (Bhat *et al.*, 2020).

In 2010, a research effort was initiated to specifically inform the MOA for the two tumor sites (mouse small intestine, rat oral cavity) observed in the 2-year bioassay on Cr(VI) (Thompson *et al.*, 2011a). At the time, it was anticipated that transcriptomic analyses would provide significant insight into the MOA, perhaps without the need for more “traditional” toxicity and genotoxicity assays. However, understanding the MOA through transcriptomics alone proved challenging and the more traditional studies have proved invaluable for informing the MOA, including *in vivo* micronucleus studies in the intestine (O’Brien *et al.*, 2013; Thompson *et al.*, 2015b) and *in vivo* mutation assays in the small intestine and oral cavity (Aoki *et al.*, 2019; O’Brien, *et al.*, 2013; Thompson *et al.*, 2015c, 2017c). One possible explanation for the limited ability of transcriptomics to inform the MOA more precisely is that synchrotron-based X-ray fluorescence (XRF) microscopy has revealed that chromium does not distribute uniformly within the intestinal mucosa; chromium fluorescence in transverse intestinal sections of mice exposed to 180 ppm Cr(VI) for 90 days was primarily detected in the intestinal villi with little or no fluorescence in the crypts (Thompson *et al.*, 2015a). As such, measuring transcriptomic responses in samples containing differentiated villus enterocytes exposed to chromium and proliferating crypt enterocytes with minimal or no chromium exposure has likely complicated the interpretation of transcriptomic data.

The purpose of the present study was to analyze gene expression in intestinal sections of mice exposed to 0.1–180 ppm Cr(VI) in drinking water for 7 or 90 days from a previously conducted bioassay (Thompson *et al.*, 2011b) using formalin-fixed paraffin-embedded (FFPE) tissues. Unlike previous studies that employed whole-genome microarray analysis of homogenized duodenal samples (Kopec *et al.*, 2012a,b; Rager *et al.*, 2017), the present study used RNA sequencing following microdissection of crypt and villus regions from FFPE transverse duodenal sections (Figure 1). Tissue fractionation allows for the determination of differential responses in two functionally distinct regions of the intestinal mucosa that receive vastly different levels of Cr(VI) exposure. Not only does this allow for investigation of dose-responses to Cr(VI) in the two compartments, but also allows for better understanding of how the normal crypt and villus transcriptome differences change as a function of chromium dosimetry and histopathological evidence of toxicity. These results are further discussed in the context of MOA analysis and the human health risk assessment of Cr(VI). In addition, the fractionated data are broadly compared with the previously published unfractionated results from the same animals.

**Figure 1.**
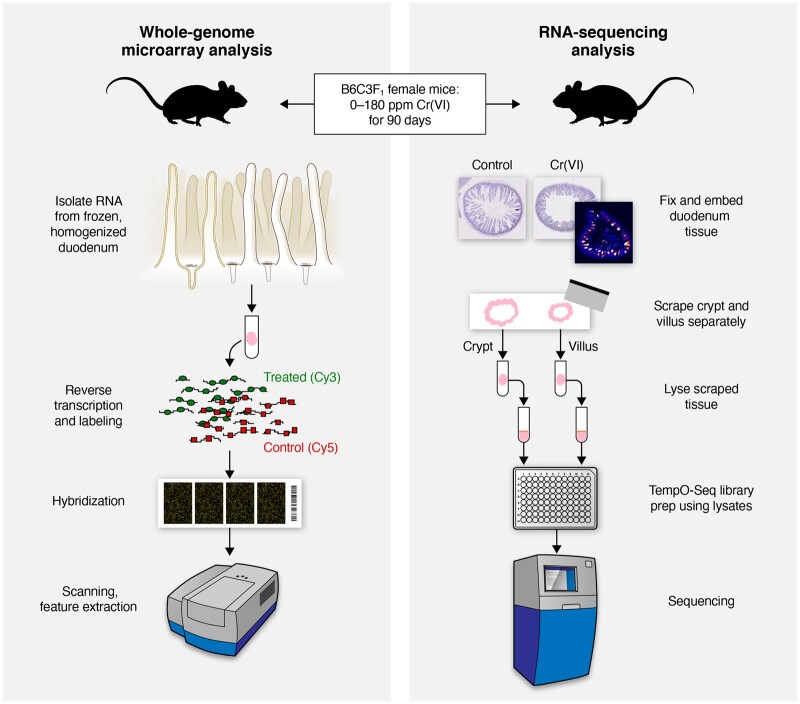
Schematic detailing and comparing the sample preparation and study design included in this study (right) and previous studies (left) by Kopec *et al*. (2012a,b) and Rager *et al.* (2017).

## MATERIALS AND METHODS

###  

####  

##### Animal treatment and tissue preparation

Animal husbandry, study design, and test substance information have been previously described in detail (Thompson *et al.*, 2011b). Briefly, female B6C3F_1_ mice (Charles Rivers Laboratories International, Inc.) were exposed to Cr(VI), as sodium dichromate dihydrate, in drinking water (*ad libitum*) at concentrations ranging from 0.3 to 520 mg/l. The estimated doses across the two exposure durations are shown in Table 1. After 7 and 90 days of exposure (referred to herein respectively as days 8 and 91), mice were euthanized by CO_2_ anesthesia and exsanguination. Small intestines were fixed in 10% neutral-buffered formalin, embedded in paraffin, and sections approximately 5–6 µm in thickness were mounted to slides to be used for RNA sequencing.

**Table 1.** Estimated Doses of Cr(VI) Intake Based on Drinking Water Concentrations^*a*^

Drinking Water Concentration, ppm Cr(VI)	Cr(VI) Intake, mg/kg bw/day	
	Day 8	Day 91
0	0.0	0.0
0.1	0.03	0.02
1.4	0.4	0.3
5	1.2	1.1
20	4.9	4.6
60	13.1	11.6
180	30.4	31.0

a Source: Thompson *et al.* (2012).

##### RNA sequencing

Two 5-µm FFPE unstained transverse duodenal sections from each mouse (*n* = 5 per group, for a total of 70 mice) were mounted on a glass slide (uncovered). The crypt region was physically removed (via microdissection) and discarded from one histologic section, and the villus removed and discarded from the adjacent section, by an American College of Veterinary Pathologists (ACVP)-certified anatomic pathologist (J.C.W.) at Experimental Pathology Laboratories (Sterling, Virginia). The remaining mounted villus and crypt tissues, respectively, were then processed individually (total of 140 samples) at BioSpyder Technologies (Carlsbad, California) by scraping the sections from the slides and processing the tissue according to the TempO-Seq protocol, a targeted RNA sequencing technology, as previously described (Yeakley *et al.*, 2017). Briefly, messenger RNA (mRNA) targets were hybridized with a detector oligomer probe mix, made up of 30 146 probes spanning the mouse whole transcriptome (each gene has 1–3 probes). Nuclease digestion of excess oligomers was conducted, followed by ligation to result in a pool of amplification templates sharing polymerase chain reaction (PCR) primer “landing sites.” Following PCR (at which point successful library preparation was confirmed for all samples by sufficient amplification), clean-up, and pooling, DNA libraries from each sample were sequenced (single-end, 50 bp read length) using a HiSeq 2500 Ultra-High-Throughput Sequencing System (Illumina, San Diego, California).

##### Data processing and analysis

Sequencing data were analyzed using packages in the R software environment, version 3.5.2 (cran.r-project.org/). The number of sequenced reads per probe was extracted from FASTQ files generated from the sequencing experiment, with each probe representing a gene-specific sequence. Samples with an overall sequencing depth (total reads across all probes) lower than two standard deviations below the mean sequencing depth across all samples, or with gene diversity (total number of genes sequenced) lower than two standard deviations below the mean number of genes sequenced per sample were excluded from the comparative analysis. Count data from all samples that passed this sequencing depth quality criterion were used for further comparative analyses.

##### Identification of genes with significant differential expression across concentrations

The DESeq2 R package (v128.1) (Love *et al.*, 2014) was used to normalize data such that sample-to-sample variation in sequencing depth was considered. Statistical methods within DESeq2 were used to calculate fold-change and identify differentially expressed genes (DEGs) associated with exposure by conducting statistical comparisons between groups that share a characteristic (Love *et al.*, 2014). In this study, the various treatment groups were compared with controls of the same sex. Differentially expressed probes (DEPs) were defined as those with a false discovery rate (FDR) < 10% for any chosen comparison between treatment groups, based on *p* values adjusted for multiple testing using the Benjamini and Hochberg (BH) procedure (Love *et al.*, 2014). Unique DEGs were identified from respective DEPs.

##### Benchmark dose analysis

Dose-response modeling was conducted using the BMDExpress software (v2.2) (Phillips *et al.*, 2019). Normalized expression data for all samples as generated using DESeq2 were loaded into BMDExpress without transformation, using probe IDs from the TempO-Seq experiment as gene identifiers. A Williams trend test (with *p* value cutoff = .05) was used to identify genes altered by exposure to Cr(VI). No fold-change filters or correction for multiple tests were applied. Benchmark dose (BMD) analysis was conducted using the following models: linear, power, hill, 2° and 3° polynomial, and exponential models 2–5. The models were run assuming constant variance and a benchmark response (BMR) of one standard deviation. Functional classification was conducted using the gene set collections available within the BMDExpress software (Reactome gene sets), based on significantly dose-responsive genes (ie, genes with a winning model fit *p* value ≥ .1), and removing genes according to the default parameters as follows: genes with BMD/BMDL > 20, BMDU/BMDL 40, BMDs above 5 mg (highest dose), and/or genes with a BMD > 10-fold below the lowest positive dose. No filters for minimum or maximum number of genes per gene set were applied. BMDs for the gene sets were also calculated. Additional settings for the BMD modeling and pathway/signaling analyses can be found in the Supplementary Materials.

##### Identification of pathway-level alterations across concentrations

Biological pathways that were associated with the transcriptomic response profiles were identified using the DESeq2 results. For genes for which multiple probes were used to measure expression, the probe with the highest sequencing count across all samples was selected as the representative gene to be used in the pathway analyses. Mouse gene identifiers were converted into human identifiers, when available, using the R package biomaRt (v2.38.0) based on the Ensembl genome database (http://uswest.ensembl.org/index.html). Gene expression data were then queried for enrichment of gene sets within the canonical pathway (CP) subcollection (c2.cp.v7.4) available through the Molecular Signatures Database (MSigDB: http://software.broadinstitute.org/gsea/msigdb/index.jsp, last accessed June 2021), which includes gene sets from several pathway databases (eg, the BioCarta online maps of metabolic and signaling pathways [BIOCARTA]; Nishimura, 2001), the Kyoto Encyclopedia of Genes and Genomes (KEGG; Ogata *et al.*, 1999), the Pathway Interaction Database (Schaefer *et al.*, 2009), and the Reactome database of reactions, pathways, and biological processes (REACTOME; Jassal *et al.*, 2020)).

Enrichment of gene sets and pathways was evaluated by two methods: the first follows the analysis employed by the gene set enrichment analysis (GSEA) platform made available by the Broad Institute (http://software.broadinstitute.org/gsea/index.jsp), the second employed a more simple hypergeometric test for overrepresentation. The GSEA method (Subramanian *et al.*, 2005) determines whether sets of genes (eg, the constituents of a molecular signaling pathway) are significantly concordant between various defined groups (in the case presented herein, different doses) based on a ranking metric (in this case, the statistical measure of differences in expression between treated and control mice, using the Wald statistic as determined with DESeq2). The GSEA statistical method was applied within the Platform for Integrative Analysis of Omics data (PIANO) R package (v1.22.0) (Varemo *et al.*, 2013). Gene set enrichment significance was calculated using permutation-based nominal *p* values based on weighted Kolmogorov-Smirnov test enrichment scores and adjusted for multiple hypothesis testing by calculating FDRs using the BH method (Subramanian *et al.*, 2005). For the hypergeometric test, all DEGs for each treatment group (ie, an FDR of < 10% as described above) were tested for overrepresentation among the gene sets in the CP subcollection using the Fisher combined probability test function within the PIANO package. For both methods, the minimum and maximum gene set size (number of member genes in the gene set that are present in the list of genes measured in the TempO-Seq experiment, and thus have gene-level statistics) was set to 5 and 500, respectively (defaults to 1 and infinity), and gene sets with an FDR < 10% were considered significantly enriched.

##### Data availability

RNA sequencing data are publicly available at NCBI’s Gene Expression Omnibus (https://www.ncbi.nlm.nih.gov/geo/) (GEO series accession number GSE182812).

## RESULTS

To examine exposure effects of Cr(VI) on gene expression in mouse duodenum, RNA sequencing was performed on duodenal crypt and villus samples from female mice exposed to tap water or 0.1–180 ppm Cr(VI) in the drinking water for 7 or 90 days. All sample libraries passed quality control requirements for sequencing. Following sequencing, five samples were removed from the analysis due to low sequencing depth and/or low gene diversity (criteria described in Materials and Methods section). Each failed sample was from an animal in a different treatment group (ie, no two failed samples were from the same dose and timepoint).

**Figure 2.**
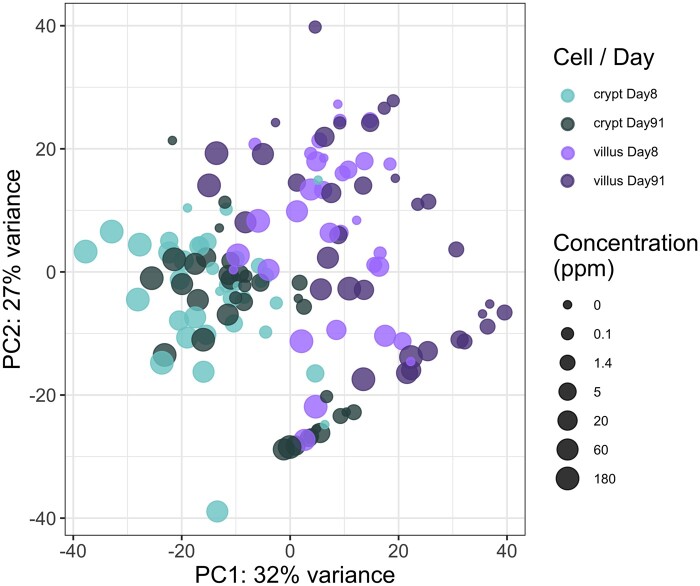
Principal components analysis plot of all samples included in the analysis. The different tissue compartments and timepoints are represented by color (see legend), and the size is scaled by the concentration of hexavalent chromium (increasing size for increasing concentration).

The variance in transcriptomic profiles across the entire dataset was visualized using principal components analysis (PCA). As can be seen in Figure 2, the PCA demonstrated that the samples varied most between the tissue compartments, next by dose, and varied minimally by duration of treatment.

###  

#### Confirmation of Tissue Compartment Isolation

**Figure 3.**
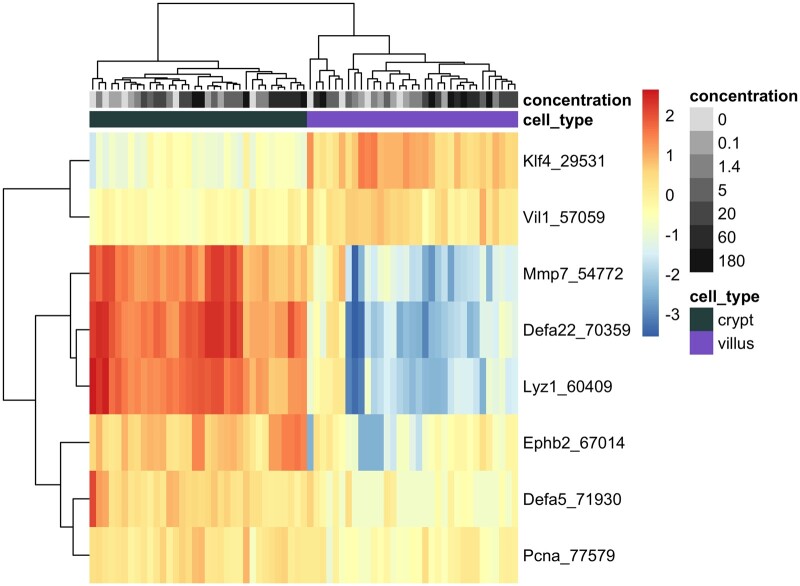
Heatmap of selected genes representing markers of crypt and villus cells at day 91 across all dose groups. Normalized expression level per sample per gene is represented by the distance of each individual sample from the mean expression level for that gene across all samples. Unsupervised clustering according to Euclidean distances is demonstrated by the ordering of genes (rows) and individual tissue samples (columns), with distances depicted by dendograms. Color and grayscale identifiers are assigned to each sample (columns) across the top of the heat map according to tissue compartment (green for crypt, purple for villus) and concentration of hexavalent chromium (grayscale increasing in darkness with increasing concentration).

The isolation of crypt and villus tissue compartments from the FFPE duodenal sections was confirmed by investigating the mRNA expression levels of crypt- and villus-specific markers in untreated and treated animals, with a focus on the basal level expression (ie, untreated animals). For example, the expression of the proliferating cell nuclear antigen DNA synthesis gene was significantly higher in crypt samples relative to villi in untreated animals, as was the expression of several genes specific to Paneth cells (secretory epithelial cells located in the small intestinal crypts; Mariadason *et al.*, 2005; Wang *et al.*, 2011), such as the matrix metalloproteinase 7 (*Mmp7*), defensin, alpha 5 and 22 (*Defa5* and *Defa22*), lysozyme 1 (*Lyz1*), and ephrin type-B receptor 2 (*Ephb2*; Figure 3). In the villus compartment, Krüppel-like factor 4 (*Klf4*), a transcription factor expressed in differentiated epithelial cells, and Villin-1 (*Vil1*), a regulator of intestinal epithelial cell morphology, were higher when compared with crypt specimens in time-matched untreated animals (Figure 3 and Supplementary Table 1). These compartment-specific differences were more significant at day 91 compared with day 8 of vehicle treatment, indicating that the difference in gene expression profile increases over time/age. Cr(VI) exposure did not have a significant effect on the compartment-specific differences in these genes (Figure 3). Similar mRNA changes have been reported previously using tissue fractionation from fresh intestinal tissue and microarray analysis of gene expression (Mariadason *et al.*, 2005).

#### Transcriptomic Changes Associated With Exposure to Cr(VI)

**Figure 4.**
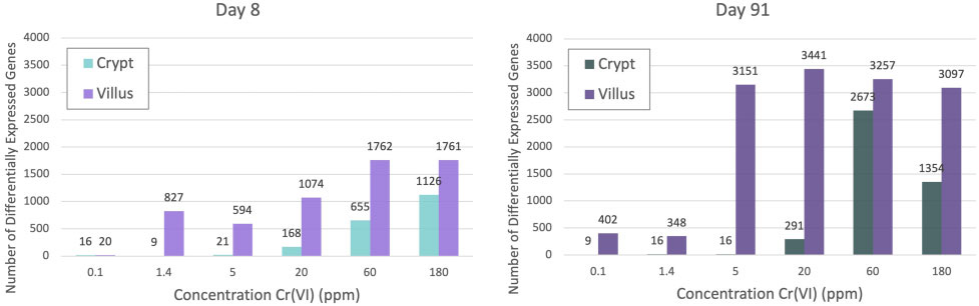
Bar charts showing the number of differentially expressed genes per treatment group in each tissue compartment at days 8 and 91.

The transcriptomic response to Cr(VI) varied in the crypts and villi at both time points (Table 2 and Figure 4). There was very minimal transcriptomic response at both days 8 and 91 in the crypt at concentrations < 20 ppm (4.9 and 4.6 mg Cr(VI)/kg bw/day for days 8 and 91, respectively), with 21 or fewer DEGs at either timepoint (full DESeq2 analysis results for treatment effect can be found in Supplementary Table 3). The DEGs in each of these low-dose groups were nearly identical. For example, the same 16 genes were differentially expressed at day 91 for both 1.4 and 5 ppm, whereas six out of the nine DEGs at 0.1 ppm were represented within these 16 DEGs. The response in the crypt, thus, appears to exhibit a nonlinear (perhaps hockey stick) dose-responsive increase in DEGs. In the villi, the number of DEGs was substantially higher at all concentrations relative to the number of DEGs in the crypts at both timepoints (with the exception of 0.1 ppm at day 8), and qualitatively appear to follow a more linear dose-responsive trend at day 8 and a sigmoidal shape at day 91 (Figure 4). This higher level of response at the molecular level aligns with the higher level of exposure to Cr(VI) in the villi relative to the crypts (Thompson *et al.*, 2015a,b). Many DEGs that were specific to the villi appear to be related to a cellular stress response (discussed further in Enrichment Analysis of Individual Dose Groups section below). For example, the dual oxidase 2 (*Duox2*) gene, which can be induced by pathologic changes such as inflammation (Burgueno *et al.*, 2019), was significantly increased in the villi at day 91 at all concentrations except for 1.4 ppm relative to the time-matched controls, while *Duox2* was unchanged in crypts at day 91 at any concentration. The total number of DEGs in each compartment generally followed a dose- and time-responsive increase, the possible exception being villi at day 91. Although there is a time component, the number of DEGs in the villi plateaued at approximately 3000 at ≥ 5 ppm (Figure 4).

**Table 2.** Number of Significantly DEGs^*a*^ in Each Group

		Drinking Water Concentration, ppm Cr(VI)					
		0.1	1.4	5	20	60	180
		C(VI) dose, mg/kg bw/day^*b*^					
		0.02–0.03	0.3–0.4	1.1–1.2	4.6–4.9	11.6–13.1	30.4–31.0
Crypt	Day 8	16	9	21	168	655	1126
	Day 91	9	16	16	291	2673	1354
Villus	Day 8	20	827	594	1074	1762	1761
	Day 91	402	348	3151	3441	3257	3097

a Defined by comparison to control mice at the same timepoint with BH-adjusted *p* value < .1 (ie, FDR <10%).

b mg/kg bw/day shown in ranges to represent the estimated Cr(VI) dose across the 2 timepoints (see Table 1).

**Table 3.** Number of Significantly Dose-Responsive Probes and Genes for Each Tissue Compartment and Timepoint

		Day 8	Day 91
Crypt	Probes	3135	5212
	Genes	2556	4714
Villus	Probes	2329	4724
	Genes	2085	4040

**Table 4.** Top 5 Most Significantly Enriched Gene Sets by Functional Classification Within BMD Analysis (Defined by Lowest *p* Value From Fisher’s Exact Two-Tail Test Using Significantly Dose-Responsive Genes as Determined by BMDExpress) for Each Tissue Compartment at Each Timepoint

GO/Pathway/Gene Set/Gene Name	Fisher’s Exact 2- Tailed	BMD Median (mg/kg bw/day)
Crypt—day 8		
Metabolism of RNA	8.58E-19	16.59
mRNA splicing—major pathway	4.98E-15	16.72
mRNA splicing	2.66E-14	16.72
Processing of Capped Intron-Containing Pre-mRNA	4.22E-13	16.59
Metabolism	1.05E-12	16.38
Crypt—day 91		
Metabolism of RNA	1.26E-30	8.53
Signaling by GPCR	2.73E-27	7.04
GPCR downstream signaling	9.50E-27	7.57
G alpha (s) signaling events	1.49E-22	14.43
Olfactory Signaling Pathway	2.51E-21	18.85
Villus—day 8		
Translation	2.97E-17	13.84
GTP hydrolysis and joining of the 60S ribosomal subunit	3.80E-13	10.64
Eukaryotic translation initiation	5.02E-13	9.91
Cap-dependent translation initiation	5.02E-13	9.91
SRP-dependent cotranslational protein targeting to membrane	1.05E-12	11.67
Villus—day 91		
G alpha (s) signaling events	1.76E-25	7.21
Olfactory signaling pathway	1.49E-22	20.54
GPCR downstream signaling	4.69E-17	2.92
Signaling by GPCR	8.57E-17	3.22
Metabolism of RNA	2.10E-14	3.10

**Figure 5.**
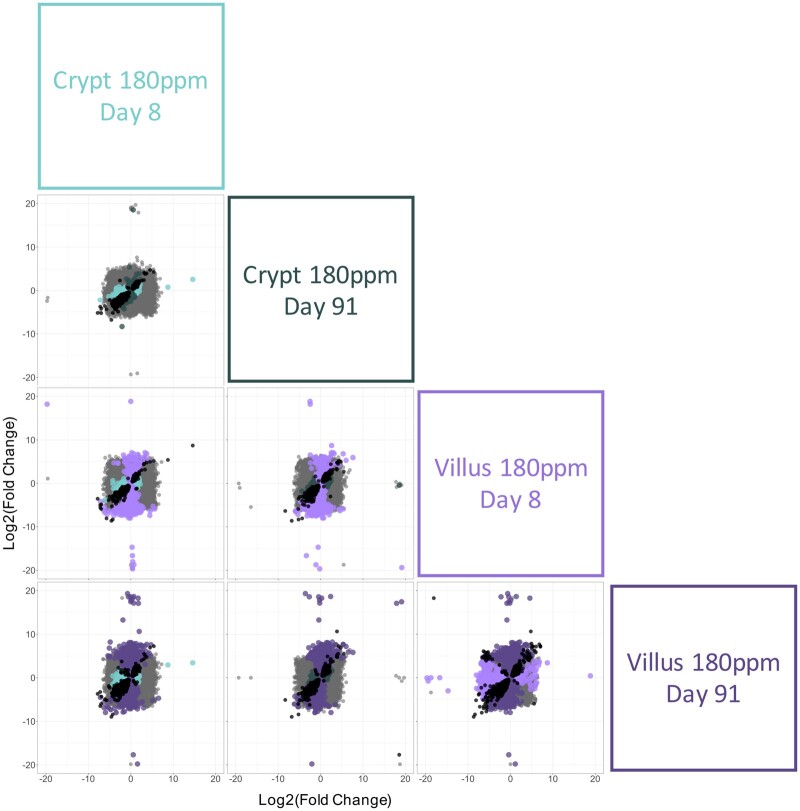
Comparison of differentially expressed genes (DEGs) associated with exposure to 180 ppm hexavalent chromium in the crypt and villus. The fold change (exposed/control) in mRNA level for all genes are plotted on the *x*- and *y*-axes according to the labels in the boxes on the right side of the matrix. Significant DEGs (relative to controls) are shown in different colors by tissue compartment type (green for crypt, purple for villus). All of the genes that were significantly differentially expressed (relative to controls) by both treatments in a given comparison are represented by black points in each plot. All genes that were not significantly differentially expressed in either treatment group are represented by gray points.

In addition to differences in the number of genes that were differentially expressed across Cr(VI) concentrations and tissue between compartments, the profile of the individual DEGs was also compared between groups to understand the overlap (and lack thereof) of DEGs between the compartments. At 180 ppm Cr(VI), the DEGs within the crypts at days 8 and 91 had a high degree of overlap, as well as similarity in the magnitude of fold change, as evidenced by the proportionality of the black points in the upper left corner of Figure 5. In the villus compartment, however, there were many more DEGs that were exclusive to one timepoint or the other (dark vs light purple points in lower right corner of Figure 5) or that were significantly altered in the opposite direction (cross pattern of black points in the lower right corner of Figure 5) at each timepoint, demonstrating that time was a more influential factor in the villi versus the crypts. Among the different compartments, there was a strong compartment difference in DEGs at day 8 as evidenced by the large number of green and purple points (middle left square in Figure 5). At day 91 there was significant overlap in DEGs (black points) in crypts and villi and, of course, many more significantly altered genes in the villi (purple points in Figure 5).

#### Benchmark Dose Modeling of Gene Expression Data

**Figure 6.**
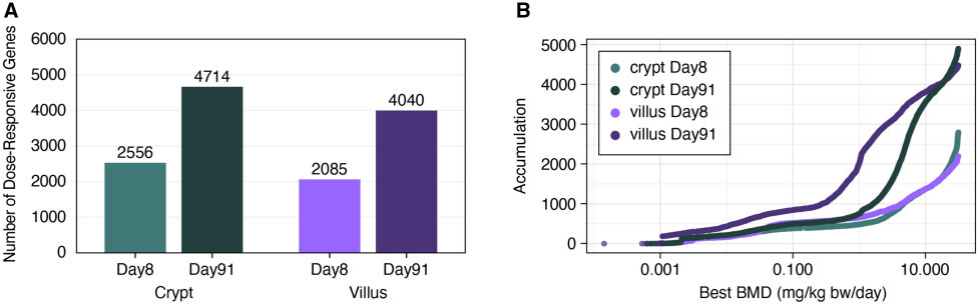
A, Number of significantly dose-responsive genes (fit *p* value ≥ .1) per tissue compartment and timepoint. B, Accumulation plot of best benchmark doses among significantly dose-responsive probes.

The dose-response across all individual probes was analyzed using BMD modeling. The many dose groups used in this study enabled an especially informative assessment of dose-responsive, cell type-specific transcriptomic changes. A total of 19 419 probes across the four groups (crypt and villus at days 8 and 91) were included for BMD modeling following the prefilter step (Williams Trend test *p* value < .05) of the normalized data from DESeq2 for all probes. Among those that passed the prefilter step, 15 400 were significantly dose-responsive, as determined by a winning model fit *p* value ≥ .1 (Supplementary Table 3). When probes were collapsed to individual genes, a total of 13 395 genes were significantly dose-responsive (Table 3 and Figure 6A). Of these, 8409 were nonredundant. As shown in Figure 6B, there tended to be more genes/probes with high BMD values at day 8 than day 91 as evidenced by the large leftward shift in accumulation plots from days 8 to 91. Within a given timepoint, more genes/probes had higher BMD values in the crypts than villi, as evidenced by the different heights of the accumulation curves, as well as the leftward shift in the day 91 villus accumulation plot relative to the day 91 crypt accumulation plot (Figure 6B).

#### Functional Classification of Dose-Responsive Genes

Functional classification (ie, enrichment of signaling pathways) of the dose-responsive genes was analyzed and visualized within the BMDExpress software. The top-most significantly enriched gene sets among dose-responsive genes were related to RNA metabolism, splicing, and signaling involving G-protein coupled receptors (GPCR), which was evident in both tissue compartments at both time points, with the topmost statistically significant enrichment of these gene sets being observed in the crypt at day 91 (Table 4). The enrichment of these transcriptional signaling gene sets was driven by upregulated genes within the gene sets, and was more significant at day 91 than at day 8 (ie, lower adjusted *p* value according to Fisher’s Two-Tailed test). The enrichment of these gene sets demonstrates a nonspecific stress response, with these gene sets being related to a variety of other signaling pathways or changes in gene expression. The significantly enriched gene sets with the lowest median BMD values were in the villus at day 91 and included: Sialic acid metabolism (downregulated genes, 0.16 mg/kg bw/day), HDMs [histone demethylases] demethylate histones (upregulated genes, 0.62 mg/kg bw/day) and Drug-mediated inhibition of ERBB2 signaling (downregulated genes, 0.67 mg/kg bw/day; Table 5).

**Table 5.** Gene Sets With the Lowest 5 BMD Median Values Among the Significantly Enriched Gene Sets by Functional Classification Within BMD Analysis (Defined by *p* Value < .1 From Fisher’s Exact Two-Tailed Test Considering Significantly Dose-Responsive Genes as Determined by BMDExpress) for Each Tissue Compartment at Each Timepoint Sialic acid metabolism

GO/Pathway/Gene Set/Gene Name	Fisher’s Exact 2-Tailed	BMD Median (mg/kg bw/day)
Crypt—day 8		
Eukaryotic Translation Elongation	0.09	1.74
Chylomicron clearance	0.09	2.51
Biosynthesis of maresins	0.003	3.05
Mitochondrial translation elongation	0.06	3.21
Mitochondrial translation termination	0.07	3.21
Crypt—day 91		
Chemokine receptors bind chemokines	1.31E-04	1.60
Utilization of ketone bodies	0.05	2.65
PERK [protein kinase R (PKR)-like endoplasmic reticulum kinase] regulates gene expression	0.02	2.75
OAS [oligoadenylate synthetase] antiviral response	0.01	2.78
Glutathione conjugation	0.08	2.92
Villus—day 8		
Pyrimidine catabolism	0.04	1.34
Butyrophilin family interactions	0.09	1.95
Gluconeogenesis	0.04	2.64
Nucleotide salvage	0.005	2.89
Eukaryotic Translation Elongation	0.004	2.95
Villus—day 91		
0.08	0.16	
HDMs demethylate histones	0.01	0.62
Drug-mediated inhibition of ERBB2 signaling	0.01	0.67
Nonhomologous end-joining	0.002	0.67
DDX58/IFIH1-mediated induction of interferon-alpha/beta	0.06	0.67

**Figure 7.**
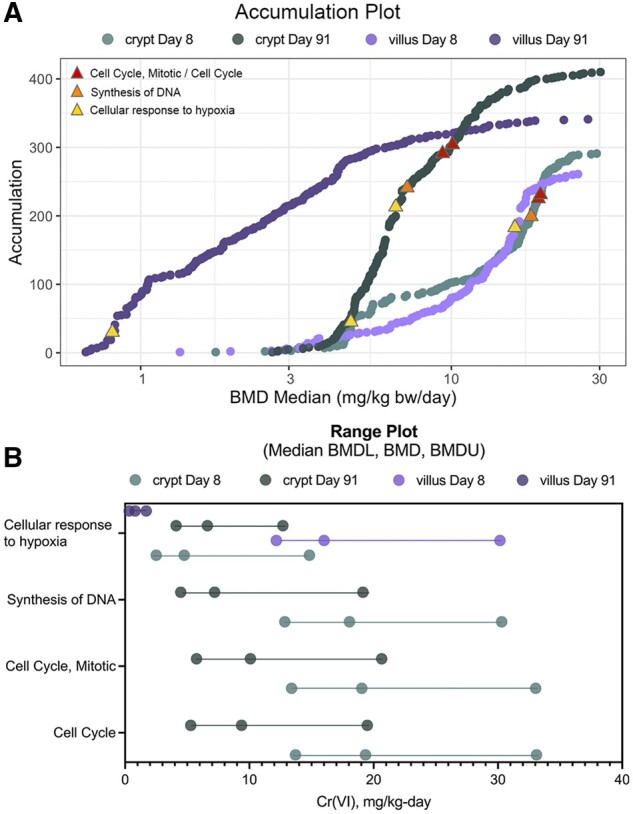
BMDExpress analysis visualizations. A, Accumulation plots for enriched pathways for all four treatment groups, with select gene sets discussed herein annotated by red, orange, and yellow triangles as indicated in the inset legend. B, Range plots for the same selected gene sets depicted by triangles in (A).

The BMD results demonstrated a prominent increase in the expression of genes related to cell cycle. For example, several gene sets related to cell cycle were significantly enriched in the crypt samples at day 91, including “Cell Cycle,” “Cell Cycle, Mitotic,” and “Mitotic G1 phase and G1/S transition” (median BMDs ranging from 6.03 to 10.09 mg Cr(VI)/kg bw/day, which corresponds to a concentration between 20 and 60 ppm Cr(VI)) (Figure 7 and Supplementary Table 4). The enrichment of these gene sets related to cell cycle was a prominent signal among dose-responsive genes for the crypt, with such gene sets representing some of the most statistically significantly enriched. There was less enrichment of such cell cycle-relevant gene sets in the villus, although enrichment was evident for some gene sets specific to mitosis (eg, “Regulation of mitotic cell cycle,” “Mitotic anaphase”).

**Figure 8.**
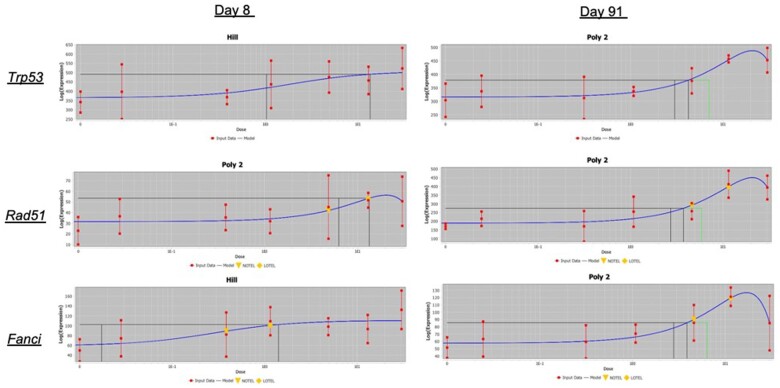
Dose-response curves for genes involved in DNA replication and/or DNA repair. The winning model curves are shown for Trp53, Rad51, and Fanci at days 8 and 91 in the crypt.

Because the role of DNA damage in the MOA for Cr(VI)-induced tumors is of interest (see Discussion section), the individual DEGs driving enrichment of gene sets related to DNA damage response and/or DNA replication at the high concentrations were investigated, as some genes in these gene sets are also related generally to cell cycle and DNA replication (eg, genes that are part of the Ubiquitin-Proteasome System; Abbas and Dutta, 2017). Genes that are known to be related to DNA damage that were significantly dose-responsive included: *Fanci* (Fanconi Anemia Complementation Group I), *Rad51* (RAD51 Recombinase), and *Tp53* (Tumor protein 53), all significantly upregulated at days 8 and 91. The dose-response for these genes in the crypt at days 8 and 91 are shown in Figure 8. Except for *Fanci* at day 8, all of these had relatively high BMDL_1SD_ values. Functionally, *Tp53*, which is responsive to diverse cellular stresses and can induce cell cycle arrest, apoptosis, senescence, DNA repair, and/or changes in metabolism, is related to cell cycle changes both dependent and independent of DNA damage. At day 91, the gene sets “Cell cycle checkpoints” and “G1/S DNA Damage Checkpoints” were both highly significantly enriched among dose-responsive genes, including *Trp53* (Supplementary Table 4). *Rad51* is similarly related to cell proliferation, is upregulated in proliferating cells, and is presumably involved in DNA repair that is related to DNA replication (Yamamoto *et al.*, 1996). The Fanconi anemia pathway is activated by hypoxic stress (Scanlon and Glazer, 2014), which aligns with the upregulation of markers of hypoxic stress (eg, significant increase in the hypoxia-inducible factor-alpha [*Hif1-a*] gene in the crypt at high concentrations, and in the villus at nearly all concentrations of Cr(VI) (Supplementary Table 2). Gene sets related to hypoxic stress and Hif1-a signaling were enriched in the crypts among dose-responsive genes: “Regulation of gene expression by Hypoxia-inducible Factor” (median BMD 10.94 mg/kg bw/day) and “Cellular response to hypoxia” (median BMD 6.62 mg/kg bw/day) at day 91. “Cellular response to hypoxia” was not enriched in the crypt at day 8, whereas this gene set was enriched in the villus at day 8 (median BMD 16.01 mg/kg bw/day), but not at day 91. Hypoxic stress has been previously observed in the small intestine of mice orally exposed to Cr(VI), including at the gene expression level (Chappell *et al.*, 2019).

Overall, BMD modeling demonstrated upregulation of nonspecific transcriptional and translational signaling in both tissue compartments, with a stronger (more significant) signal at day 91 compared with day 8. Upregulation of gene sets related to cell cycle signaling was the next most prominent theme among dose-responsive genes, in the crypt. A more subtle increase in cell cycle signaling was evident in the villus.

#### Enrichment Analysis of Individual Dose Groups

Although the BMD modeling offered valuable information regarding the biology of dose-responsive transcriptional changes, GSEA was also conducted for each treatment group relative to controls. As described in the Materials and Methods section, pathway analysis was conducted using the GSEA platform made available by the Broad Institute as well as by the simpler hypergeometric test. The top-most significantly enriched pathways were similar across the two methods and were also similar to gene sets described previously using BMD modeling (Supplementary Tables 5 and 6). In agreement with the BMD results, the transcriptomic response at the gene set level occurs at lower concentrations of Cr(VI) in the villi than in the crypts (using either method for gene set enrichment). In fact, the small number of DEGs in the crypt at ≤ 20 ppm at days 8 and 91 precluded pathway enrichment analysis using the hypergeometric test (ie, no gene sets were enriched by this method). The genes that were differentially expressed at low concentrations were related to growth response induced by mitogenic stimulation (*Egr3*, up), response to inflammation and tissue injury (*Saa1*, down), pancreatic-related genes (*Reg2*, expressed specifically in the small intestine, down; *Ppy*, down), production of the mucous barrier in the gut lumen (*Muc6*, down), cell adhesion and signal transduction (*Ctnnd1*, down), intestinal bile acid uptake (*Slc10a2*, down), phosphate transport (*Slc34a2*, up), iodide and monocarboxylate transport (*Slc5a8*, up), and immunoglobulins and T-cell receptor enhancers (both directions). Notably, all of these genes have generally very low expression independent of treatment, and did not pass the prefiltering step in the BMD modeling analysis—suggesting an absence of dose-response in the low and high dose regions. A notable exception was *Ctnnd1*, which had relatively high average expression, was elevated at ≥ 60 ppm, and was dose-responsive in the crypt at 90 days exhibiting a BMD of 3.51 mg/kg bw/day or between 5 and 20 ppm (see Table 1) (Fit *p* value = 0.52, Polynomial 2 model).

It was of particular interest to examine the pathway enrichment at 0.1, 1.5, and 5 ppm Cr(VI) in the crypt to understand if the low number of DEGs in these dose groups converged in any signaling pathways or functional processes. The small number of gene changes resulted in a complete lack of enrichment by the hypergeometric analysis. Using the GSEA method, which allows for a broader view of enrichment by accounting for relatively subtle expression changes, gene sets related to zinc homeostasis, translation, ribosome, and response to metal ions were downregulated, and gene sets related to immune and inflammatory response (eg, interferon signaling) were upregulated Supplementary Table 5. Beginning at 5 ppm, gene sets related to DNA replication were significantly enriched (up direction; GSEA method).

At 20 ppm, the modest number of DEGs in the crypt resulted in only 34 gene sets enriched by the hypergeometric test (adjusted *p* value < .1), which were all in the down direction (Supplementary Table 6). These gene sets included xenobiotic metabolism, proteasome degradation, nuclear receptors, and antigen processing. At ≥ 60 ppm Cr(VI), gene sets related to nuclear receptors, fatty acid metabolism, peroxisome proliferator-activated receptor signaling, P450 oxidation/xenobiotic metabolism, and glucuronidation were all enriched in a downregulation direction. Upregulated enriched gene sets were related to transcription and translation, cell cycle and DNA replication (including mitotic checkpoints), and some DNA damage response gene sets that are related to cell cycle and chromosome maintenance were also enriched at day 91.

Overall, the gene set enrichment in the crypt demonstrated an increase in transcription and translation across concentrations at day 8, and an increase in cell proliferation signaling at the high doses at both days 8 and 91. Decreases in lipid metabolism and xenobiotic metabolism, as well as in normal intestinal cell processes (ie, digestion and absorption) were evident at day 91 across all concentrations, and at days 8 and 91 at the highest concentrations.

Within the villus compartment, it was of particular interest to examine the pathway enrichment at 0.1 and 1.4 ppm Cr(VI) at day 91 prior to the approximately 10-fold increase in DEGs beginning at 5 ppm (see Figure 4). There was no significant enrichment according to the hypergeometric test for these groups, indicating that the DEGs are not constituents of signaling pathways or gene sets. According to the GSEA method (ie, not strictly limiting to DEGs), gene sets related to ribosomal processes, translation, defensins, response to metal ions, and amino acids metabolism were all enriched, driven by downregulated DEGs. With the exception of responses to metal ions, the same gene sets were not enriched at 5 ppm. Some gene sets related to translation were enriched for upregulated genes at higher concentrations, beginning at 60 ppm, at day 91. These results suggest that the genes altered at concentrations below 5 ppm in the villus appear to have minimal relationship to the dose-responsive signal in this tissue compartment, related to Cr(VI) exposure.

Similar to the crypt samples, “Cellular Response to Hypoxia” was enriched, only at 180 ppm day 8. Downregulation of nuclear receptors and metabolic gene sets was also evident at the highest 2 concentrations at both days 8 and 91 (eg, Reactome “Nuclear receptor transcription pathway,” WikiPathways “Nuclear receptors metapathway,” Reactome “Peroxisomal lipid metabolism,” and WikiPathways “Oxidation by cytochrome P450”; Supplementary Tables 5 and 6). The Reactome gene set “Cellular Response to Chemical Stress” was significantly enriched in the up direction at the highest concentrations at both days 8 and 91.

A single DNA damage gene set (Reactome “G1 S DNA damage checkpoints”) was enriched in the villus at 5, 20, and 180 ppm at day 91 according to the hypergeometric method (adjusted *p* value < .1). This gene set was also enriched according to the GSEA method, only at 180 ppm at day 8 (adjusted *p* value < 0.1). No other gene sets with “DNA damage” in the title were enriched in the villus at any timepoint or Cr(VI) concentration. The enrichment of this gene set was due exclusively to genes encoding proteasomal subunits. In contrast to the crypts, a very minimal signal for upregulation of DNA replication or cell cycle gene sets in the villus was observed according to either enrichment method, and were exclusive to the highest concentrations and day 91, with the exception of some such gene sets enriched at the highest concentration of 180 ppm at day 8.

Overall, the gene set enrichment in the villus demonstrated a cellular stress and damage response, including an increase in translation at higher concentrations and a decrease in defensins in the lower concentrations. In contrast to the crypt, the villus did not have a strong cellular proliferation signal (Supplementary Tables 5 and 6).

#### Comparison to Previous Microarray Results From Homogenized Duodenal Samples From the Same Study

Because transcriptomics analyses have been previously conducted on frozen duodenal tissues from animals in the same study as discussed herein, we compared the tissue compartment-specific results to gene expression results from earlier studies without separation of crypt and villus (Kopec *et al.*, 2012a; Rager *et al.*, 2017; Table 6). Similarities were apparent in the top-most enriched canonical pathways and/or individual genes across these studies, including cellular stress and injury (eg, Hif1a, Mapk, and Eif signaling), cellular proliferation, oxidative stress genes, GPCRs, lipid and oxidative metabolism, and DNA replication/DNA repair signaling. This study provides additional information regarding tissue compartment specificity (or lack thereof) of some of these signals; for example, DNA replication and repair response and Hif1a signaling were evident at a similar significance level in both compartments, whereas the cellular proliferation signaling and apoptosis were much more significant in the crypt compartment than the villus, according to functional classification following BMD modeling.

**Table 6.** Comparison in Gene Set Enrichment and Dose Responsiveness Across Transcriptomic Studies at Day 91 According to BMD Modeling Analyses

	Results From Previous Work	Results from Present Study	Phenotypic Anchoring
Cellular stress and injury	Rager *et al.* (2017): EIF2 signaling, Hif1-a signaling, mTOR signaling, MAPK signaling, Apoptosis signaling, Death receptor signaling Kopec *et al.* (2012a,b)	Crypt: Hif1a signaling, EIF2 signaling, oxidative stress, apoptosis signaling, cellular responses to stress, and response to hypoxia Villus: Hif1a signaling, EIF2 signaling, oxidative stress, response to hypoxia, and DNA Damage/Telomere Stress-Induced Senescence	Villus atrophy and blunting, crypt proliferation
Cellular proliferation	Rager *et al.* (2017): EIF2 signaling, EIF4 and p70S6K signaling, mTOR signaling, RAN signaling	Crypt: EIF2 signaling, cell cycle, mitotic cell cycle, DNA replication, and G2/M checkpoints Villus: EIF2 signaling, mitotic anaphase and metaphase, and DNA repair	Crypt hyperplasia and crypt elongation
Cancer	Rager *et al.* (2017): Molecular mechanisms of cancer		No evidence of preneoplastic or neoplastic lesions at day 8 or 91
Dose-response	Kopec *et al.* (2012a,b): Virtually no DEGs at ≤ 5 ppm Rager *et al.* (2017): No DEGs at ≤ 5 ppm	Crypt: < 20 DEGs at ≤ 20 ppm Cr(VI) Villus: sharp increase in DEGs at ≥ 5 ppm Cr(VI)	Crypt hyperplasia observed at ≥20 ppm^*a*^

a Based on reanalysis of Thompson *et al.* (2011a,b,c) in Cullen *et al.* (2016).

## DISCUSSION

Transcriptomic analyses designed to inform the MOA for mouse small intestine tumors in Cr(VI) studies have been previously conducted. In 2011, we published histopathological, dosimetry, and molecular results from a 90-day Cr(VI) drinking water study that included the same Cr(VI) concentrations as in the NTP (2008) 2-year cancer bioassay, as well as two additional lower concentrations (Thompson *et al.*, 2011b). Frozen duodenal tissues collected from animals in the aforementioned study were analyzed for transcriptomic changes using Agilent whole-genome oligonucleotide microarrays (Kopec *et al.*, 2012a,b). These data were subsequently reanalyzed and compared with select *in vitro* Cr(VI) data (Rager *et al.*, 2017). Refinements in the analysis presented herein relative to previous analyses include: (1) whole transcriptome RNA-Seq technology versus microarray hybridization techniques; (2) analysis conducted in the same tissue blocks used to assess histopathology versus across cohorts in the study, thereby improving phenotypic anchoring; (3) analysis in crypts and villi separately versus homogenized tissue, enabling a better understanding of compartment-specific effects; and (4) omission of fold-change filtering (eg, previous application of a 2-fold change minimum for individual genes criterion) for the inclusion of genes into BMD modeling and functional classification, allowing for the identification of subtle treatment effects in the intestine. These refinements further inform the MOA and provide additional insight into the potential risk that environmental/background levels of Cr(VI) pose with regard to oral cancer risk.

**Figure 9.**
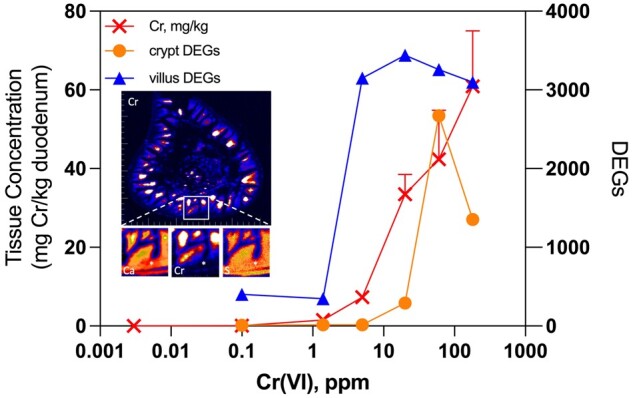
Integration of duodenal transcriptomic analyses with duodenal chromium (Cr) dosimetry data. The left axis and red crosses depict duodenal Cr levels measured in mice exposed to hexavalent chromium (Cr(VI)) for 90 days using ICPMS (see Kirman *et al.*, 2012). The right axis depicts the number of differentially expressed genes in the crypt and villus at day 91 reported in the present study. The inset depicts X-ray fluorescence (XRF) maps of Cr in a transverse duodenal section from a mouse exposed to 180 ppm Cr(VI) for 90 days (larger image), with magnification of mucosa (yellow square) and XRF maps of calcium (left smaller box below main image), Cr (middle), and sulfur (S, right) (white asterisks mark the crypt compartment). Note: the transcriptomic data plotted here, the tissue Cr data, and XRF mapping are all from a single animal study originally described in Thompson *et al.* (2011a,b).

At a high level, the transcriptomic responses align closely with duodenal Cr levels measured in the same underlying study (Thompson *et al.*, 2011b; Figure 9). Significant increases in duodenal Cr levels measured by inductively coupled plasma mass spectrometry (ICPMS) were detected in mice exposed to ≥ 5 ppm Cr(VI), whereas a nonsignificant increase was detected in mice exposed to 1.4 ppm. Duodenal levels in mice exposed to 0.1 ppm Cr(VI) (0.056 ± 0.015 mg/kg tissue) were very similar to levels in mice exposed to tap water (0.017 ± 0.007 mg/kg tissue). Notably, the approximately 3-fold difference in tissue concentration from background Cr(VI) levels (≤ 0.003 ppm) to 0.1 ppm Cr(VI) resulted in 402 DEGs in the villus and only 9 DEGs in the crypt after 90 days of exposure. A further 10-fold increase in exposure to 1.4 ppm Cr(VI) resulted in an approximately 10-fold increase in tissue concentration (1.5 ± 0.27 mg/kg tissue), but did not further increase DEGs in the villus (348 DEGs). Although crypt DEGs increased from 9 to 16 from 0.1 to 1.4 ppm Cr(VI) (following 90 days of exposure), there was no increase in the number of DEGs in the crypt at 5 ppm Cr(VI) (ie, the same 16 genes were differentially expressed at both 1.4ppm and 5 ppm). In contrast, the 5-fold increase in exposure from 1.4 to 5 ppm increased the number of DEGs in the villus from 348 to 3151. Taken together, these data indicate potential thresholds in response to Cr(VI), especially in the crypt compartment (which is also evident at day 8). Although the increased number of DEGs in the crypt at ≥ 20 ppm might indicate Cr beginning to reach the crypt compartment, XRF microscopy in transverse duodenal sections of mice exposed to 180 ppm Cr(VI) for 90 days indicate little or no exposure to the crypt compartment (Figure 9). This XRF mapping coupled with the transcriptomic analyses presented herein indicates that the crypt compartment is essentially unaffected by Cr(VI) exposures ≤ 5 ppm for up to 90 days. Although crypt hyperplasia was noted in mice at 5 ppm in the NTP (2008) cancer bioassay, that pathology occurred after two years of exposure and was without evidence of neoplasia in mice exposed to 5–20 ppm Cr(VI). The absence of tissue dosimetry and molecular responses to Cr(VI) indicate that Cr(VI) levels below the current maximum contaminant level of 0.1 ppm do not pose a cancer risk. Notwithstanding the evidence for thresholds in response, an important consideration in the MOA and risk assessment for Cr(VI)-induced intestinal cancer in mice is the potential role of genotoxicity. *In vivo* genotoxicity studies have revealed the absence of micronucleus formation (O’Brien *et al.*, 2013; Thompson *et al.*, 2015b), mutations (Aoki *et al.*, 2019; Thompson *et al.*, 2015c, 2017c), γ-H2AX activation (Thompson *et al.*, 2015b), or preneoplastic lesions in the rodent target tissues (small intestine and oral cavity) following exposure to ≤ 180 ppm Cr(VI) for 7, 28, or 90 days (NTP, 2007, 2008; Thompson *et al.*, 2011b, 2012, 2015b, 2017b). Aside from the lack of genotoxicity, there is clear histopathological and transcriptomic evidence for dose-dependent increases in crypt hyperplasia. Morphologic evidence stems from subjective differences in crypt proliferation by hematoxylin and eosin (H&E) staining (NTP, 2007, 2008; Stout *et al.*, 2009; Thompson *et al.*, 2011b), as well as objective measures in proliferation such as increases in crypt enterocyte number and crypt length (Thompson *et al.*, 2015a,b). Molecular evidence for proliferation in the form of transcriptomic responses was observed herein, as well as in previous microarray-based analyses conducted on homogenized duodenal samples (Kopec *et al.*, 2012a; Rager *et al.*, 2017).

Because of the apparent lack of *in vivo* genotoxicity in the duodenum and clear evidence of increased cell proliferation, transcriptomic responses should be interpreted in the context of these data. Phenotypic anchoring of *Fanci* and *Rad51* focused on potential nongenotoxic explanations for their alteration. Rad51 protein expression has been reported in low levels in the crypt regions of untreated mice but not villus regions (Yamamoto *et al.*, 1996). *In vitro* analyses have shown that *Rad51* mRNA levels in serum-stimulated synchronized cells increase as cells progress from G_0_ through S-phase and in mitogen- and lipid polysaccharide-stimulated cultured lymphocytes (Yamamoto *et al.*, 1996). Thus, while the dose-dependent increase in *Rad51* expression in the crypt might be interpreted as concern for DNA damage, it is more consistent with histopathological evidence for crypt hyperplasia in the absence of evidence for micronucleus formation and increased mutation frequency. The Fanconi anemia pathway, including FANCI, is activated by acute severe hypoxic stress *in vitro* (Scanlon and Glazer, 2014), which aligns with the gene expression changes in other markers of hypoxic stress (eg, significant increase in the *Hif1-a* gene in the crypt at high concentrations, and in the villus at nearly all concentrations of Cr(VI); Supplementary Table 2). Gene sets related to *Hif1a* signaling and hypoxic stress were enriched in the crypt and the villus among dose-responsive genes at day 90. Relatedly, gene-level indicators of hypoxia-induced oxidative stress were demonstrated in the duodenum of Cr(VI)-exposed mice in another oral exposure study (Chappell *et al.*, 2019).

Due to the fact that many genes active in DNA damage repair are also active during cell replication (Zannis-Hadjopoulos and Rampakakis, 2011), transcriptomic evidence for or against genotoxicity is difficult to parse, and activation of genes and pathways involved in both cell proliferation and DNA damage response in tissues actively undergoing proliferation presents a challenge in interpretation. For example, *Rpa1* and *Rpa3* (Replication protein A isoforms), which are involved in DNA replication and repair (Oakley and Patrick, 2010) were significantly dose-responsive in the villus at day 91; however, *Rpa1* was significantly downregulated, whereas *Rpa3* was upregulated, further complicating the clarity of the role of the Cr(VI) effect on these replication proteins in the villus. The expression of *Rpa* genes was not altered by Cr(VI) exposure in the crypt compartment. A previous microarray analysis compared the *in vivo* transcriptomic responses in the duodenum with *in vitro* data from peer-reviewed literature and publicly available high-throughput screening (HTS) data from the Tox21 database (Rager *et al.*, 2017). Therein, it was demonstrated that Cr(VI) activated p53-related signaling pathways *in vitro* but not *in vivo*. In the current analysis, DNA damage response and/or DNA replication pathways were enriched at the high concentrations and were largely composed of genes related to cell cycle and DNA replication. Genes known to be related to DNA damage included *Fanci*, *Rad51*, and *Tp53* were upregulated in the crypt compartment (Figure 8). With regard to comparisons of *Tp53* results with those in Rager *et al.* (2017) who required a ≥ 2-fold change in gene expression, the BMD modeling herein indicates that the maximum fold change in *Tp53* was approximately 1.5-fold (Supplementary Tables 1 and 5). Notably, *Tp53* is related to cell cycle increase both dependent and independent of DNA damage (O’Brien *et al.*, 2013). Further, recognized downstream p53 targets *Gadd45a* and p21 (*Cdkn1a*) were not induced in any group, with the exception of *Cdkn1a* in the crypt at 180 ppm at day 91. Although there may be hundreds of gene targets of p53 (Fischer, 2017), none of the approximately 50 genes within the KEGG p53 signaling pathway (KEGG PATHWAY: p53 signaling pathway; https://www.genome.jp/pathway/hsa04115) associated with “DNA repair and damage prevention” were upregulated in this study, at any dose or either timepoint.

Overall, the transcriptomics data do not indicate a direct genotoxic effect of Cr(VI) on the small intestine. These results are consistent with *in vivo* genotoxicity assays in the small intestine (see above) as well as new review on the mammalian *in vivo* genotoxicity of Cr(VI) that concluded that while Cr(VI) can cause *in vivo* genotoxicity in nontarget tissues following intraperitoneal and gavage dosing of high concentrations of Cr(VI), the data did not support direct genotoxicity in the mouse small intestine (Thompson *et al.*, forthcoming). One criticism often levied against *in vivo* genotoxicity assays is that they are of relatively short duration. Notably, most OECD test guideline *in vivo* genotoxicity assays recommend exposure durations of four weeks or less (eg, Test Nos. 474, 475, 488, and 489; OECD, 2016a,b,c, 2020). This is not a deficiency, but rather consistent with the objective of determining whether genotoxicity is likely to be an early key event in the MOA. In some cases, longer-term treatment durations are contraindicated due to potential confounding from clonal expansion (Lambert *et al.*, 2005; Manjanatha *et al.*, 2017; OECD, 2020).

## CONCLUSION

Previous microarray-based transcriptomic analyses of homogenized mouse duodenal tissue have supported evidence for alterations in cellular pathways and processes related to increased toxicity, cell proliferation, and DNA damage. The compartment-based transcriptomic analyses presented herein provide additional resolution for molecular responses to Cr(VI) and critical dose-response information that informs human health risk assessment of Cr(VI). Although transcriptomics alone might be interpreted as supporting genotoxicity, based on the upregulation of select genes related to DNA damage, the phenotypic anchoring to histopathology, XRF microscopy, and genotoxicity assays in the target tissues allow for refined interpretation of transcriptomic responses that are ambiguous due to their involvement in both normal cell cycle progresses as well as DNA damage responses. The minimal transcriptomic responses in the crypt below 20 ppm Cr(VI) support arguments that the toxicity and carcinogenicity of Cr(VI) are high-dose phenomena that should not be extrapolated to environmental levels. Although 5 ppm Cr(VI) resulted in increased cell proliferation in the crypt at the end of the 2-year NTP (2008) bioassay, this is not altogether surprising given the number of DEGs altered in the villus at 5 ppm (Figure 9), which was similar to the number of DEGs in the higher dose groups. The molecular responses in the villus at 5 ppm did not manifest as obvious histopathological changes at day 91; however, microscopic evaluations are subjective, as evidenced by the fact that NTP (2008) reported no evidence of villus blunting or crypt hyperplasia in rats, whereas a reanalysis found mild indications of such (Cullen *et al.*, 2016). A closer examination of the histologic sections at day 91 might reveal subtle evidence of morphologic changes in the villi, but the lack of transcript response in the crypts indicates an absence of molecular effects at day 91. Data indicate that effects in chronic assays tend to occur at approximately 5 times lower concentrations than in subchronic assays (Dourson *et al.*, 1996), so it is not surprising that crypt hyperplasia observed at 20 ppm (but absent at 5 ppm) after 90 days of exposure was observed at 5 ppm after 2 years of exposure in the NTP (2008) bioassay. Importantly, toxicity criteria based on crypt hyperplasia in the 2-year NTP (2008) bioassay with appropriate application of dose-response modeling and relevant uncertainty factors should be protective of both noncancer and cancer effects of Cr(VI) in the intestine. The absence of transcriptomic responses in the crypt at ≤ 5 ppm after 90 days of exposure provides further scientific justification for nonlinear extrapolation approaches in the development of safety criteria for oral exposure to Cr(VI). 

## SUPPLEMENTARY DATA

Supplementary data are available at *Toxicological Sciences* online.

## DECLARATION OF CONFLICTING INTERESTS AND FUNDING DISCLOSURE

G.A.C. and C.M.T. are employed by ToxStrategies, Inc., a private consulting firm that provides services to private and public organizations on toxicology and risk assessment issues. J.C.W. is employed by Experimental Pathology Laboratories, Inc., a private, employee-owned company specializing in toxicologic pathology evaluation and consultation. ToxStrategies, Inc. received consulting fees from the Cr(VI) Panel of the American Chemistry Council (ACC); the work reported in this article was conducted during the normal course of employment, and no authors received personal fees. The sponsors were provided an opportunity to review the manuscript prior to submission. The purpose of the review was for the authors to receive input on the clarity of the science presented but not on the interpretation of research results. The researchers’ scientific conclusions and professional judgments were not subject to the funders’ control; the contents of this manuscript reflect solely the view of the authors. C.T. and J.W. have presented study findings in meetings with regulators, including public meetings, on behalf of the Cr(VI) Panel of the ACC.

## Supplementary Material

kfab152_Supplementary_Data
