# Correction to: Crypt and Villus Transcriptomic Responses in Mouse Small Intestine Following Oral Exposure to Hexavalent Chromium

**DOI:** 10.1093/toxsci/kfad118

**Published:** 2023-11-17

This is a correction to: Grace A Chappell, Jeffrey C Wolf, Chad M Thompson, Crypt and Villus Transcriptomic Responses in Mouse Small Intestine Following Oral Exposure to Hexavalent Chromium, *Toxicological Sciences*, Volume 186, Issue 1, March 2022, Pages 43–57, https://doi.org/10.1093/toxsci/kfab152

Due to a syntax error in the differential gene expression analysis R code, the number differentially expressed genes (DEGs) in the crypt for the Day 91 1.4-ppm group was duplicated for the Day 91 5-ppm group resulting in the need for the following corrections: (1) the reported number of 16 DEGs in Table 2 for 5 ppm at Day 91 should be 10 DEGs; (2) the reported number of 16 DEGs in Figure 2 for 5 ppm at Day 91 should be 10 DEGs; (3) the fourth sentence in the “Transcriptomic Changes Associated With Exposure to Cr(VI)” results section should read “For example, 16 genes were differentially expressed at day 91 at 1.4 ppm, of which 6 out of the 9 DEGs at 0.1 ppm and 8 out of the 10 DEGs at 5 ppm were represented within these 16 DEGs.”; (4) the sixth sentence in the second paragraph of the Discussion section should read “Although crypt DEGs increased from 9 to 16 from 0.1 to 1.4 ppm Cr(VI) (following 90 days of exposure), there was no increase in the number of DEGs in the crypt at 5 ppm Cr(VI).”; (5) updated Supplemental Table S2. The corrected number of DEGs in the 5 ppm group continues to indicate thresholds in gene expression in the mouse crypts following prolonged exposure to hexavalent chromium.

**Revised Table 2**

**Table 2.** Number of significantly differentially expressed genes^a^ in each group

		Drinking water concentration, ppm Cr(VI)					
		0.1	1.4	5	20	60	180
		C(VI) dose, mg/kg bw/day^b^					
		0.02-0.03	0.3-0.4	1.1-1.2	4.6-4.9	11.6-13.1	30.4-31.0
Crypt	Day 8	16	9	21	168	655	1126
	Day 91	9	16	10	291	2673	1354
Villus	Day 8	20	827	594	1074	1762	1761
	Day 91	402	348	3151	3441	3257	3097

a defined by comparison to control mice at the same timepoint with BH-adjusted p-value <0.1 (i.e., false discovery rate <10%)

b mg/kg bw/day shown in ranges to represent the estimated Cr(VI) dose across the two timepoints (see Table 1).

**Revised Figure 4**

**Figure 4.**
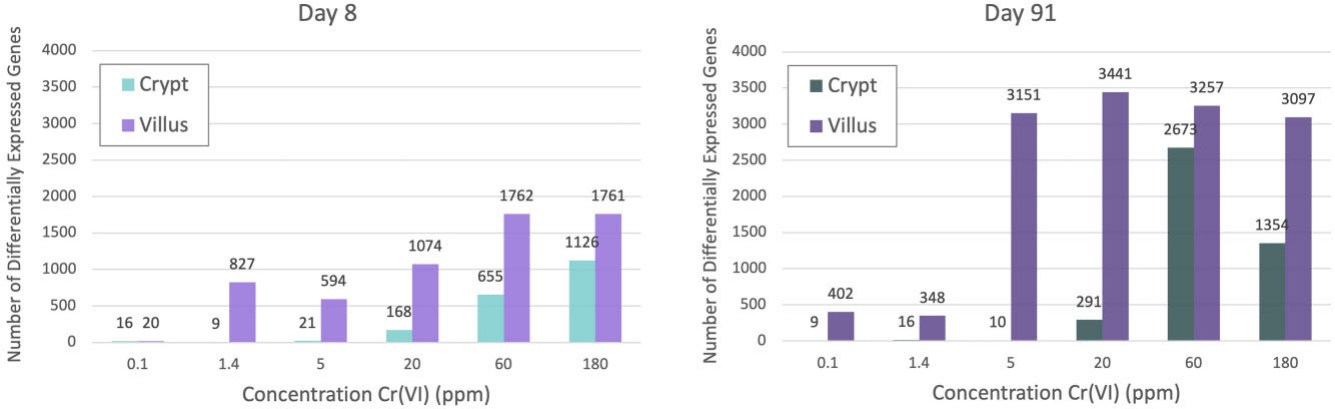


These details have been corrected only in this correction notice to preserve the published version of record.

