# Supplementary material for: Crypt and Villus Transcriptomic Responses in Mouse Small Intestine Following Oral Exposure to Hexavalent Chromium
Source: Toxicol Sci. 2021 Dec 22;186(1):43–57. doi: 10.1093/toxsci/kfab152 (PMC8883354; doi:10.1093/toxsci/kfab152)
Supplement: kfab152_Supplementary_Data [file kfab152_supplementary_data.zip › toxsci-21-0384-File011.docx]

Crypt and villus transcriptomic responses in mouse small intestine following oral exposure to hexavalent chromium

Chappell, GA^1^; Wolf, JC^2^; Thompson, CM^3^

^1^ToxStrategies, Inc, Asheville, NC

^2^EPL, Sterling, VA

^3^ToxStrategies, Inc, Katy, TX

**Supplemental Materials: BMDExpress Analysis parameters**

***Benchmark Dose Analysis***

BMDExpress2 Version: BMDExpress 2.30.0507 BETA

Operating System: Windows 10

hill version: Hill Model. (Version: 2.18; Date: 03/14/2017)

power version: Power Model. (Version: 2.19; Date: 03/14/2017)

linear version: Polynomial Model. (Version: 2.21; Date: 03/14/2017)

poly 2 version: Polynomial Model. (Version: 2.21; Date: 03/14/2017)

poly 3 version: Polynomial Model. (Version: 2.21; Date: 03/14/2017)

exponential 2 version: Exponential Model. (Version: 1.11; Date: 03/14/2017)

exponential 3 version: Exponential Model. (Version: 1.11; Date: 03/14/2017)

exponential 4 version: Exponential Model. (Version: 1.11; Date: 03/14/2017)

exponential 5 version: Exponential Model. (Version: 1.11; Date: 03/14/2017)

Models fit: hill, power, linear, poly 2, poly 3, exponential 2, exponential 3, exponential 4, exponential 5

Maximum Iterations: 250

Confidence Level: 0.95

Constant Variance: 1

BMR Type: Standard Deviation

BMR Factor: 1.0

Restrict Power: 1

Highest Dose: 30.38

Lowest Positive Dose: 0.028

Best Model Selection: Lowest AIC

Fit Selected Models with Multiple Threads: 24

Number of Available Processors On Machine: 6

Destory Model Processes If Run More Than: 60000 milliseconds

BMDL and BMDU Model Selection: Compute and utilize in best model selection

Flag Hill Model with 'k' Parameter <: 1/3 of Lowest Positive Dose

Best Model Selection with Flagged Hill Model: Select Next Best Model with P-Value > 0.05

***Signaling Pathway Analyses***

Organism Code: mmu

Signaling Pathway File Creation Date: 04/15/21

Pathway DB Name: REACTOME

Deduplicate Gene Sets: false

BMDExpress2 Version: BMDExpress 2.30.0507 BETA

Remove Promiscuous Probes: true

Remove BMD > Highest Dose from Category Descriptive Statistics: true

Remove BMD with p-Value < Cutoff: 0.1

Remove genes with BMD/BMDL >: 20.0

Remove genes with BMDU/BMDL >: 40.0

Remove genes with BMD values > N fold below the lowest positive does: 10.0

Identify conflicting probe sets: 0.5
